# The Role of Lactoferrin in Modulating Inflammation and Preventing Preterm Birth: A Narrative Review

**DOI:** 10.3390/nu17193164

**Published:** 2025-10-07

**Authors:** Alessandro Messina, Safae El Motarajji, Bianca Masturzo, Paolo Manzoni

**Affiliations:** 1 Department of Obstetrics and Gynecology, University Hospital “Degli Infermi”, 13875 Ponderano, Italy; alessandro.messina@aslbi.piemonte.it (A.M.); bianca.masturzo@aslbi.piemonte.it (B.M.); 2 Department of Public Health and Pediatrics, University of Torino, via Santena 5bis, 10126 Turin, Italy; 3 Department of Public Health and Pediatric Sciences, University of Torino School of Medicine, 10121 Turin, Italy; paolo.manzoni@aslbi.piemonte.it; 4 Division of Paediatrics and Neonatology, Degli Infermi Hospital, 10126 Ponderano, Italy

**Keywords:** lactoferrin, preterm birth, inflammation, cytokines, pregnancy outcomes

## Abstract

Background: Preterm birth (PTB) remains a leading cause of neonatal morbidity and mortality worldwide. Inflammatory cytokines, particularly IL-6, are central to PTB pathogenesis. Lactoferrin (LF), an iron-binding glycoprotein with antimicrobial and immunomodulatory properties, has been proposed as a potential protective factor against PTB. This narrative review aimed to synthesize current evidence on LF supplementation and its effects on inflammation, cytokine modulation, biochemical markers, and obstetric outcomes related to PTB. Methods: Eight clinical studies involving pregnant women at risk of PTB were included. LF was administered orally, vaginally, or through combined regimens, with variations in dosage and duration. Reported outcomes encompassed inflammatory markers, cervical and uterine parameters, oxidative stress biomarkers, and obstetric or neonatal endpoints. Results: Across the studies, LF supplementation was consistently associated with reduced pro-inflammatory cytokines, improvements in cervical length and uterine activity, and favorable changes in oxidative stress markers. Clinically, supplementation was linked with prolonged gestation, fewer preterm births, and reduced neonatal intensive care admissions. Immunological analyses further suggested a positive modulation of cytokine profiles in amniotic fluid. Conclusions: LF appears to exert multifaceted immunomodulatory effects that mitigate inflammation and support pregnancy maintenance. Although findings point to its potential role in PTB prevention, they should be interpreted with caution given the limited and heterogeneous evidence. Further large-scale, multicenter randomized trials are needed to confirm efficacy and to establish optimal dosage, route, and timing of administration.

## 1. Introduction

Preterm birth is defined by the World Health Organization (WHO) as all births occurring before 37 completed weeks of gestation or fewer than 259 days since the first day of a woman’s last menstrual period [1]. The estimated global prevalence of preterm birth in 2020 was 9.9%, corresponding to approximately 13.4 million live preterm births [2]. Of the 5.3 million deaths in children under five years worldwide recorded in 2019, about 0.94 million (17.7%) were attributed to complications of preterm birth, making it the leading cause of child mortality globally [3].

Although many socio-demographic, nutritional, medical, obstetric, and environmental factors have been linked to an increased risk of spontaneous preterm birth, its etiology remains only partially understood [4]. Inflammatory cytokines are consistently implicated as central mediators in its pathogenesis. Clinical studies have reported elevated concentrations of IL-6, IL-8, and IL-10 in amniotic fluid from women who experienced preterm labor and delivery. Among these, IL-6 is particularly important, as its marked elevation has been identified as a key marker of intrauterine infection and a potential trigger for preterm delivery [5]. In more than 30% of cases, preterm premature rupture of membranes (PROM) is associated with microbial invasion of the amniotic cavity and high levels of pro-inflammatory cytokines, especially IL-6 and IL-8 [6].

The risk of preterm birth has also been shown to correlate with increased levels of hsCRP, IL-10, IL-6, TNF-α, total cholesterol, triglycerides, and HDL (all *p* < 0.05) [7]. Notably, elevated IL-6 concentrations in amniotic fluid have been associated with intra-amniotic inflammation even in the absence of clinical signs, highlighting the need for more accurate diagnostic and therapeutic strategies [8].

Lactoferrin (LF) is an 80 kDa glycoprotein found in human milk, where it makes up 10–15% of total protein content, and in mucosal secretions such as saliva and intestinal fluids [9]. It has shown significant potential in mitigating oxidative stress across multiple biological systems [10,11]. Specifically, lactoferrin attenuates the pro-inflammatory response of neonatal monocyte-derived macrophages [10,11,12]. Treatment of LPS-activated neonatal macrophages with human lactoferrin, isolated from heparinized cord and peripheral blood, reduces cytokine production (TNF, IL-1β, IL-6, IL-8, IL-10) and decreases activation marker expression and phagocytic capacity [12].

Beyond its antimicrobial and immunomodulatory properties, lactoferrin also plays a role in iron homeostasis. By binding free iron, LF reduces its availability to pathogens while facilitating absorption in the host. This dual function makes LF relevant in preventing and treating iron deficiency anemia, as shown in pregnant populations. Importantly, oral LF does not act as a direct iron source. Instead, it modulates the IL-6–hepcidin axis, improves iron absorption, and exerts systemic anti-inflammatory effects. In contrast, vaginal LF acts locally, influencing the vaginal microbiota, mucosal immunity, and cytokine balance [11].

The biological effects of LF depend on the route of administration: oral intake influences systemic iron metabolism and inflammation, whereas vaginal administration modulates the local microbiota, mucosal immunity, and cytokines. LF is also abundant in colostrum and breast milk, where it contributes to neonatal immune defense and gastrointestinal development. These pleiotropic functions highlight LF’s versatility across physiological contexts. Taken together, lactoferrin may represent a promising candidate for improving adverse pregnancy outcomes not only due to its antimicrobial effects but also through its ability to modulate immune cell function and reduce inflammation [13].

Lactoferrin supplementation has also been studied in different clinical and physiological contexts for its role in systemic inflammation and immune support. Studies in healthy individuals, patients with type 2 diabetes, and pregnant women with iron deficiency anemia (IDA) have shown significant reductions in IL-6 and TNF-α levels after LF supplementation [12]. Although systematic reviews have examined the efficacy of lactoferrin in preventing preterm birth [14], they addressed the topic broadly, covering domains such as iron metabolism regulation in IDA, antimicrobial and immunomodulatory properties, and obstetric outcomes like preterm birth incidence and NICU admission.

While previous reviews [14] covered diverse domains, no work has specifically synthesized evidence on LF’s ability to modulate inflammatory pathways directly implicated in preterm birth (e.g., IL-6, prostaglandins, MMP/TIMP balance, oxidative stress). Moreover, the role of administration route (Oral Vs. Vaginal) and timing (Pre-Procedure Vs. Sustained supplementation) in shaping these outcomes has not been emphasized.

The purpose of this narrative review is therefore to summarize the available literature on the potential effects of lactoferrin supplementation on inflammation regulation, cytokine modulation, and other biochemical and obstetric outcomes associated with the risk of preterm birth.

## 2. Materials and Methods

This article was conceived and conducted as a narrative review, with the aim of synthesizing and critically discussing the available literature on the role of lactoferrin in modulating inflammatory mechanisms associated with the risk of preterm birth. To enhance methodological transparency and quality, the review was developed in accordance with the Scale for the Assessment of Narrative Review Articles (SANRA) framework [15], which provides established criteria for narrative reviews.

The methodology included a multi-database search, predefined inclusion and exclusion criteria, and a transparent selection process but did not involve a formal quality assessment of the studies or quantitative synthesis, as these were beyond the scope of this review.

### 2.1. Search Strategy

The literature search was conducted across three main electronic databases: PubMed (MEDLINE), Embase, and Web of Science from inception to 15 March 2025. Eligible studies were required to investigate the effect of lactoferrin administration either bovine or recombinant human on inflammatory markers, oxidative stress, or obstetric outcomes in pregnant women at risk of preterm birth, including those undergoing mid-trimester genetic amniocentesis. The search was restricted to articles published in English or Italian in peer-reviewed journals. In collaboration with an expert librarian, a comprehensive search strategy was developed using a combination of free-text terms and Medical Subject Headings (MeSH), including “lactoferrin,” “preterm birth,” “preterm delivery,” “inflammation,” “cytokines,” “oxidative stress”. Additionally, the reference lists of relevant reviews and included studies were manually screened to identify further eligible publications.

Eligibility criteria and data extraction items were defined A Priori, before initiating the screening process.

### 2.2. Inclusion Criteria

Based on the Population–Intervention–Comparison–Outcome (PICO) framework, the inclusion criteria were defined as follows: (i) pregnant women at risk of preterm birth, including those undergoing mid-trimester genetic amniocentesis. Inflammatory profiles detected at the time of amniocentesis have been shown to independently predict adverse outcomes such as preterm premature rupture of membranes [16]. (ii) Intervention: the administration of lactoferrin, either bovine (bLf) or recombinant human lactoferrin (rhLf), through oral or vaginal means as a preventive or therapeutic approach to modulate inflammatory processes during pregnancy. (iii) Comparison: studies including control groups with no treatment, placebo, or alternative treatments. (iv). Outcomes: the modulation of inflammatory markers (e.g., IL-6, TNF-α, PGE2), oxidative stress biomarkers, biochemical indicators in amniotic fluid or cervicovaginal secretions; obstetric parameters such as cervical length, preterm birth rates, gestational age at delivery, and neonatal outcomes (e.g., NICU admission, birth weight).

### 2.3. Exclusion Criteria

The exclusion criteria for this review were as follows: (i) studies not involving pregnant women at risk of preterm birth, such as studies on non-pregnant populations, postpartum women, or experimental laboratory models (In Vitro or animal studies); (ii) studies assessing substances or interventions other than lactoferrin, or studies in which lactoferrin was administered in combination with other investigational treatments, making it impossible to isolate its effects; (iii) articles not providing original research data, including case report, review articles, meta-analyses, editorials, commentaries, and conference abstracts.

### 2.4. Study Selection and Data Extraction

A total of 2140 records were identified through database searches (PubMed, Embase, Web of Science). After removing duplicates, 1300 abstracts were screened for eligibility by two independent reviewers (S.E.M. and A.M.) using the Rayyan platform. At the title/abstract screening stage, records were excluded primarily due to irrelevant populations (e.g., non-pregnant women), interventions not involving lactoferrin, or outcomes not related to inflammatory or obstetric endpoints. Full-text articles were then assessed for eligibility, with discrepancies resolved by discussion referring to predefined inclusion criteria. Consequently, 8 articles met the inclusion criteria and were included in the final qualitative synthesis. The PRISMA inspired flow diagram detailing the study selection process is provided in the Supplementary Material.

### 2.5. Data Synthesis

No formal meta-analysis was performed due to the substantial heterogeneity of the included studies in terms of design (Open-Label Vs. Controlled trials), populations (bacterial vaginosis, short cervix, amniocentesis), lactoferrin formulations (Bovine Vs. Recombinant), routes of administration (oral, vaginal, combined), dosages, and outcome measures. These differences precluded meaningful quantitative synthesis. To improve clarity despite this limitation, we organized the results in a stratified manner according to intervention type, route of administration, and clinical indication, and for outcomes reported in multiple studies (e.g., IL-6) we described the consistency in the direction of effect.

### 2.6. Safety Definitions and Reporting

Adverse events (AEs) and serious adverse events (SAEs) were classified according to the original study reports and, when possible, mapped to CTCAE categories [17]. We explicitly distinguished between “no events observed” and “no safety data provided.” Where available, information on dose ranges, exposure duration, and discontinuation rates was extracted.

## 3. Results

This review summarizes data from eight clinical studies conducted in Italy, involving several hundred pregnant women (see Table S1, Supplementary Material). Because multiple publications originated from the same research groups, the possibility of partial cohort overlap cannot be excluded, and participant totals should therefore be interpreted with caution.

The studies used different formulations of lactoferrin, including recombinant human lactoferrin (rhLf) and bovine lactoferrin (bLf). Lactoferrin was administered orally (one study), vaginally (six studies), or via a combination of oral and vaginal routes (one study). The clinical outcomes examined cover multiple pathophysiological and clinical aspects related to the risk of preterm birth. For clarity, the findings are organized into five main thematic areas: (3.1) inflammatory markers, (3.2) cervical and uterine parameters, (3.3) biochemical biomarkers and oxidative stress, (3.4) obstetric and neonatal clinical outcomes, and (3.5) immunological profile of the amniotic fluid.

Across the eight included studies, designs were heterogeneous. The majority were open-label clinical studies, one was a randomized trial [18], and one was a retrospective cohort [19]. Populations varied, including women with iron deficiency or anemia [20,21], shortened cervix [18], women undergoing mid-trimester amniocentesis [18,19,21], and women with bacterial vaginosis and prior preterm birth [19]. Comparators ranged from ferrous sulfate [20] to untreated controls or usual care. Lactoferrin was administered orally, vaginally, or in combined regimens, with daily doses between 100 and 300 mg and treatment durations from 10 days to several weeks. Sample sizes ranged from small pilot cohorts (n = 21) to larger series (n > 150).

### 3.1. Inflammatory Markers

Several studies have consistently reported a significant reduction in IL-6 levels following lactoferrin administration. Giunta et al. [20] observed a marked decrease in cervico-vaginal IL-6 after 10 days of oral rhLf treatment (1834 ± 0.4 pg/mL vs. 3466 ± 1.8 pg/mL; mean difference –1632 pg/mL; *p* = 0.005; 95% CI not reported), an effect that was notably maintained at 30 days (*p* = 0.05). Similarly, Paesano et al. [21] confirmed a significant reduction in both serum IL-6 at delivery (*p* = 0.0001) and cervico-vaginal IL-6 after 4 weeks (*p* = 0.0001) in women treated with a combination of oral and vaginal bovine lactoferrin (bLf). In alignment with these findings, Locci et al. [18] reported a substantial decrease in cervico-vaginal IL-6 levels in women treated with vaginal bLf for 21 days (5.823 pg/mL vs. 80.82 pg/mL; *p* < 0.0001). Vesce et al. [22] documented a significant reduction in amniotic IL-6 in patients who received lactoferrin 4 h prior to amniocentesis (242.3 ± 163.5 pg/mL vs. 1084.1 ± 1458.3 pg/mL; *p* = 0.03). With regard to prostaglandins, Paesano et al. (2012) reported a significant decrease in cervico-vaginal PGF2α levels (*p* = 0.0001), whereas Trentini et al. [23] found notably lower PGE2 concentrations in the treatment group (3.8 pg/mg creatinine vs. 5.3 pg/mg creatinine; *p* < 0.01).

### 3.2. Cervical and Uterine Parameters

Several studies have evaluated both morphological and functional parameters, including cervical length, uterine contraction frequency, and alterations in vaginal flora (AVF). Locci et al. [18] reported a significant increase in cervical length after 21 days of vaginal bLf treatment (median 37.6 mm [IQR 34.8–43.0] vs. 21.38 mm [range 20.0–23.5]; *p* < 0.0001; confidence intervals not reported), along with a corresponding reduction in the frequency of uterine contractions (>6 every 30 min: 9% vs. 20.3%; *p* = 0.05). An inverse correlation between IL-6 levels and cervical length was consistently identified in both groups (treated and control), with a correlation coefficient of *r* = –0.81 in the lactoferrin group (*p* < 0.0001). Giunta et al. [20] additionally reported a significant reduction in the prevalence of AVF in the rhLf-treated group (from 71% to 15% over 30 days; *p* = 0.0007), while no significant differences were observed in the incidence of funneling, which was absent in both groups.

### 3.3. Biochemical Biomarkers and Oxidative Stress

Trentini et al. [24] assessed the amniotic expression of matrix metalloproteinases (MMPs) and their inhibitors, observing a significant reduction in MMP-9 (*p* < 0.005) and TIMP-1 (*p* < 0.001), alongside a marked increase in MMP-2 (*p* < 0.0001). Consequently, the MMP-2/TIMP-2 ratio significantly increased in the vaginal bLf-treated group (*p* < 0.0001). Subsequently, Trentini et al. [24] investigated oxidative stress markers, demonstrating a significant reduction in TBARS levels (*p* < 0.0001 at 4 h; *p* < 0.05 at 12 h), a marked decrease in the oxidative stress index (OSI; *p* < 0.0001), and a 35% increase in total antioxidant capacity (TAS) compared to controls (*p* < 0.0001).

### 3.4. Obstetric and Neonatal Clinical Outcomes

Giunta et al. [20] reported that all patients in both the rhLf and control (ferrous sulfate) groups successfully delivered beyond 37 weeks of gestation. Complementing these findings, Miranda et al. [19] observed a significantly lower rate of preterm birth (<37 weeks) in women treated with vaginal bLf (25.0%) compared to controls (44.6%; *p* = 0.02). Moreover, the mean gestational age was significantly higher in the treatment group (37.7 ± 3.2 vs. 35.9 ± 4.1 weeks; *p* = 0.01), and there was a notable reduction in hospitalizations due to threatened preterm labor (45.0% vs. 70.8%; *p* = 0.04) as well as a lower rate of neonatal intensive care unit (NICU) admissions (8.3% vs. 21.5%; absolute risk difference –13.2%; *p* = 0.05; 95% CI not reported). No significant differences were found regarding chorioamnionitis, preterm premature rupture of membranes (PPROM) before 34 weeks, or neonatal birthweight.

### 3.5. Amniotic Immunological Profile

The study by Maritati et al. [25] investigated the impact of vaginal bLf administration on the cytokine profile of amniotic fluid. Significant reductions in various pro-inflammatory cytokines were observed, including IL-9, TNF-α, IP-10, IFN-γ, IL-1α, IL-15, and MCP-3 (*p* < 0.05–0.001). Conversely, significant increases were observed in cytokines and growth factors such as IL-17, FGF-basic, GM-CSF, G-CSF, MCP-1, and SDF-1α (*p* < 0.001). While these changes may reflect shifts in the local immune environment, it is important to note that IL-17 is typically considered a Th17-associated cytokine with context-dependent effects that can also be pro-inflammatory. Therefore, these findings should be interpreted with caution, and their clinical significance remains uncertain.

### 3.6. Safety, Adverse Events and Compliance

Safety and compliance reporting across the included studies was limited and inconsistent. Where described, no serious adverse events related to lactoferrin were observed, and only occasional mild gastrointestinal symptoms were reported [21]. In several studies no safety information was provided, while others explicitly stated the absence of side effects or complications. A few dropouts were reported, generally unrelated to treatment [18]. Overall, the available evidence suggests good tolerability of lactoferrin, but the scarcity and heterogeneity of data prevent a robust assessment of its safety profile.

## 4. Discussion

This narrative review highlights consistent evidence supporting lactoferrin (LF), in both bovine (bLF) and recombinant human (rhLF) forms, as an immunomodulatory and anti-inflammatory molecule in the prevention of adverse obstetric outcomes, particularly preterm birth and PPROM. Unlike prior reviews, our synthesis explicitly links compartment-specific inflammatory markers (amniotic, cervico-vaginal, serum) with clinical endpoints and emphasizes the role of administration route and timing. This pathway-to-outcome perspective provides clinicians with a mechanism-informed framework to interpret the potential role of LF in preterm birth prevention.

The data analyzed are consistent with the pathophysiological mechanisms underlying inflammation mediated preterm labor. Infection and inflammation-associated PPROM are initiated by the upregulation of pro-inflammatory cytokines, which modulate the expression of matrix metalloproteinases (MMPs) in amniochorionic cells [26,27,28,29]. Intra-amniotic infection or inflammation detected at the time of amniocentesis has been shown to be independently associated with subsequent PPROM in women with intact membranes and preterm labor [16]. This supports the inclusion of studies involving women undergoing amniocentesis in this review, as these cohorts provide a direct window into inflammatory mechanisms that precede and predict adverse outcomes.

At the fetal level, systemic cytokine responses characterized by elevated plasma IL-6 levels >11 pg/mL have been associated with imminent onset of spontaneous labor in patients with PPROM [30]. This offers robust evidence for the active role of pro-inflammatory cytokines in triggering preterm birth, as the elevation of fetal IL-6 precedes rather than follows the clinical onset of labor [6]. These findings align with the reviewed studies, which reported reductions in IL-6 at serum, amniotic, and cervico-vaginal levels following LF administration. This suggests a potential early interruption of the inflammatory cascade.

In parallel, LF appears to favorably influence the downstream proteolytic cascade of inflammation by modulating the expression and activity of MMPs, key enzymes involved in collagen degradation and weakening of the amniochorionic membranes. MMP-9 levels and MMP-9. TIMP ratios are significantly higher in preterm births compared to term pregnancies (*p* < 0.001), while TIMP-1 and TIMP-2 are significantly lower (*p* = 0.002 and *p* < 0.001, respectively), with mean reductions of 11% and 22% [16,31]. Comparisons of placental mRNA expression of MMP-1, MMP -2, and MMP -9 between term and preterm labor also show significantly elevated expression in the latter, indicating a molecular environment conducive to premature membrane rupture [32]. Consistently, Trentini et al. [22] reported a significant reduction in MMP-9 after intravaginal bLF, along with selective regulation of other relevant enzymes and inhibitors.

Beyond anti-inflammatory and anti-proteolytic effects, LF has been associated with improvements in cervical and uterine parameters, indirectly contributing to pregnancy stabilization [21]. bLF administration has been shown to increase cervical length, reduce uterine contraction frequency, and produce an inverse correlation between IL-6 levels and cervical length [18]. Oxidative stress, often elevated in inflammatory pregnancy conditions, also appears to improve with LF [33]. Trentini et al. [23] reported reductions in TBARS, an increase in total antioxidant capacity (TAS), and a decrease in the oxidative stress index (OSI) after intravaginal bLF administration.

Clinically, these effects translate into lower preterm birth rates, higher gestational age at delivery, fewer hospital admissions for preterm labor, and reduced NICU admissions [19,20]. A comparative study by Pino et al. [34] also provided insights into dose–response effects of vaginal LF for bacterial vaginosis, a condition linked with chronic inflammation and preterm birth risk. Women treated with 200 mg/day for 10 days showed more sustained improvements in vaginal microbiota than those treated with 100 mg/day. In the 200 mg group, 92.8% achieved a Nugent score <7 at treatment completion and 85.7% reached ≤3, with stability maintained two weeks after discontinuation. These findings suggest a dose-dependent effect of LF in restoring vaginal eubiosis, supporting the use of 200 mg/day where stronger antimicrobial and immunomodulatory action is needed [35].

Systemic administration data are also encouraging. In a four-month follow-up, daily oral LF at 100 mg twice daily reduced asymptomatic bacteriuria (7.5% vs. 30.1%; *p* < 0.00001) and acute cystitis (0.9% vs. 9.6%; *p* < 0.00001), while increasing negative urine cultures (47% vs. 15.1%; *p* < 0.00001) [36]. These results confirm LF’s efficacy as a multifactorial preventive strategy. As noted in the Methods, no meta-analysis could be performed due to study heterogeneity, which remains a limitation.

Timing of administration also seems to affect LF efficacy. Rosa et al. [37] showed that oral cbLF taken before meals produced greater reductions in serum IL-6 and improved hematological parameters. This effect may reflect partial degradation of LF by gastric proteases during meals, reducing bioavailability. Thus, dose, route, and timing are all critical factors for LF’s therapeutic effectiveness.

Finally, safety remains an important consideration. Reporting on safety, compliance, and interactions was limited and inconsistent. Where described, LF was generally well tolerated, but the scarcity of data constrains interpretation. Future trials should incorporate systematic monitoring of adverse events and adherence.

## 5. Limits

This narrative review has several limitations. First, all included studies were conducted in Italy. This reduces the generalizability of the findings to other healthcare systems and socio-cultural contexts and underscores the need for multicenter and international studies. In addition, the concentration of research within one country, and sometimes within related research teams, entails a potential risk of partial cohort overlap. This cannot be excluded based on the available reporting. For this reason, the total number of participants should be interpreted with caution, as we recalculated it conservatively to avoid double counting.

The results should therefore be interpreted with caution and regarded primarily as hypothesis-generating, to be tested in larger and more rigorous future studies. International evidence remains scarce and is mostly limited to case reports or small experimental studies. The overall methodological quality of the included studies was also limited. Most were small open-label trials, with only one randomized, controlled trial [18] and one retrospective cohort study [19]. Some used unconventional comparators, such as ferrous sulfate instead of placebo. None were multicenter trials, reducing external validity and reproducibility.

Potential funding bias also cannot be excluded, as some interventions were based on formulations provided by pharmaceutical companies. These factors collectively reduce the robustness and generalizability of the available evidence. Finally, although some studies reported positive effects, the lack of high-quality RCTs with rigorous methodology and adequate power highlights the need for further robust investigations before drawing definitive conclusions.

Finally, as only studies published in English and Italian were included, the possibility of language bias cannot be excluded.

## 6. Conclusions

The findings of this review suggest that lactoferrin supplementation, administered orally and/or vaginally, may exert multifaceted modulatory effects on inflammatory processes, biochemical markers, and immunological profiles associated with preterm birth risk. Evidence indicates that lactoferrin can reduce pro-inflammatory cytokines such as IL-6 across different biological compartments and improve cervical and uterine parameters relevant to pregnancy maintenance. Its influence on proteolytic enzymes and oxidative stress markers may also contribute to the stabilization of fetal membranes. Clinically, supplementation was associated with trends towards prolonged gestation, lower rates of preterm labor, and fewer neonatal intensive care admissions.

These results highlight the potential therapeutic role of lactoferrin in modulating pathways underlying preterm birth. However, they should be interpreted with caution, given the limited number and quality of available studies. Further large-scale, multicenter, and international randomized controlled trials are needed to confirm these findings and to better define the optimal dosage, route of administration, and timing of supplementation.

## Data Availability

The original contributions presented in this study are included in the article/Supplementary Material. Further inquiries can be directed to the corresponding author(s).

## Supplementary Materials

The following supporting information can be downloaded at: https://www.mdpi.com/article/10.3390/nu17193164/s1, Table S1. Study Characteristics.

## Abbreviations

The following abbreviations are used in this manuscript:

AF	Amniotic Fluid
bLF	bovine lactoferrin
BV	bacterial vaginosis
ID	Iron deficiency
IDA	iron deficiency anemia
LF	lactoferrin
LPS	Lipopolysaccharide
MMP	matrix metalloproteinase
NICU	Neonatal Intensive Care Unit
OSI	Oxidative Stress Index
OxS	Oxidative Stress
PGE2	Prostaglandin E2
PPROM	Preterm Premature Rupture of Membranes
PTB	preterm birth
PTD	preterm delivery
rhLf	recombinant human lactoferrin
TAS	Total Antioxidant Status
TIMP	Tissue inhibitor of metalloproteinase
