# Supplementary material for: The Role of Lactoferrin in Modulating Inflammation and Preventing Preterm Birth: A Narrative Review
Source: Nutrients. 2025 Oct 7;17(19):3164. doi: 10.3390/nu17193164 (PMC12526437; doi:10.3390/nu17193164)
Supplement: Supplementary file 1 [file nutrients-17-03164-s001.zip › nutrients-3871862-supplementary.pdf]

Table S1. Characteristics of Included Studies.

**Author (Year) Country**	**Study Type**	**Aim**	**Sample size**	**Intervention**	**Control**	**Type of lactoferrin/ treatment**	**Results**	**Safety/Compliance**	**Overlap notes**
Giunta et al. [18] (2011) Italy	Open-label double-blind pilot trial study	To evaluate the impact of recombinant human lactoferrin on the prevention of preterm delivery	21 women at 26-32 weeks pregnant, suffering from IDA, at risk of preterm delivery	**Group B**: 14 patients received 100 mg of oral rhLf twice a day before meals, for one month	**Group A**: 7 patients received 520 mg of ferrous sulfate (Ferrous Sulfate) once a day	rhLf	**AVF at baseline**: No significant differences between the two groups, with 71% of AVF in both the group, p = NR<br><br>**AVF after 10 days:** No differences between the two groups, (64% of AVF vs 57% of AVF rispectively), p = NR<br><br>**AVF after 30 days:** A significant reduction was observed in the Group B vs Group A (15% of AVF vs 57%), p = 0.0007<br><br>**IL-6cv at baseline (pg/ml)**: no differences between the two groups, (3.538 pg/ml ± 2.2 pg/ml vs 3.287 pg/ml ± 1.5 pg/ml), p = 0.821<br><br>**IL-6cv after 10 days (pg/ml):** A reduction in IL-6cv levels was observed in the Group B vs Group A (1.834 pg/ml ± 0.4 pg/ml vs 3.466 pg/ml ± 1.8 pg/ml), p = 0.005<br><br>**IL-6cv after 30 days (pg/ml):** A reduction in IL-6cv levels was	**No safety data provided**	Publications from the same research group; potential partial overlap of participants cannot be excluded

							observed in the Group B vs Group A (1.998 pg/ml ± 1.2 pg/ml vs .532 pg/ml ± 2.1 pg/ml), p = 0.05<br><br>**IL-6s detectable:** IL-6s was not detectable in 11 patients in the Group B and in 5 patients in the Group A, p = NR<br><br>**Cervical length at baseline:** Cervical length ranged from 26–32 mm in the Group B and from 24–35 mm in the Group A, p = NR<br><br>**Funneling:** No cases of funneling were observed in either group, p = NR<br><br>**Pregnancy outcome:** normal pregnancy course in both groups, with delivery at term (>37 weeks): (266 ± 9 days (Group A) vs 258 ± 4 days (Group B), p = NR		
Paesano et al. [19]<br><br>(2012)<br><br>Italy	Open-label cohort and subcohort study	To evaluate the effectiveness of combined oral and intravaginal administration of bLf in preventing PTD in pregnant women	163 pregnant women at second and third trimester of gestation<br><br>**Cohort:** Pregnant women with singleton physiological pregnancies, iron	**Group B (cohort)**: 161 pregnant women treated with oral administration of 100 mg of bLf 20% iron-saturated 2 times a day before meals, for at least 4 weeks until delivery	**Group A (subcohort)**: 11 pregnant women treated with oral administration of 100 mg of bLf 20% iron-saturated 2 times a day before meals and intravaginal tablet containing 100 mg of lyophilized bLf 20% iron saturated, every 8 h for 4 weeks of	LF	**Serum IL-6 at delivery (pg/mL:** reduction in group B at delivery, p = 0.0001<br><br>**Cervicovaginal IL-6 after 1 weeks (pg/mL):** decreased in the Group A, but remained higher than Group B, p=NR	**No side effects observed** - Maternal gastrointestinal tolerance and local vaginal safety were monitored, with no fetal or neonatal adverse outcomes (Apgar and AFI within normal range)	

			deficiency (ID) or iron deficiency anemia (IDA)<br><br>**Subcohort:** Pregnant women with singleton physiological pregnancies, iron deficiency (ID) or iron deficiency anemia (IDA)<br><br>with a confirmed threat of preterm delivery (PTD) not related to infections.		gestation, no longer than 37th week of gestation		**Cervicovaginal PGF2α after 1 weeks (ng/mL):** decreased in the Group A but remained higher than Group B, p=NR<br><br>**Cervicovaginal IL-6 after 4 weeks (pg/mL):** decreased in the Group A vs Group B, p = 0.0001<br><br>**Cervicovaginal PGF2α after 4 weeks (ng/mL):** decreased in the group A vs Group B, p = 0.0001<br><br>**Fetal fibronectin (fFN):** levels in Group A remained below 50 ng/mL at both 1- and 4-week assessments.<br><br>**Cervical lenght**: no shortening or dilation observed after 1 and 4 weeks in Group A.		
Locci et al. [22]<br><br>(2013)<br><br>Italy	Randomised, prospective, longitudinal study	To evaluate the role of bLf in modulating cervico-vaginal IL-6 levels and cervical length in pregnant women at risk of preterm delivery due to short cervix	128 asymptomatic patients at 20 – 24 weeks' gestation with singleton ongoing pregnancy	**Group B:** 64 patients received 300 mg of LF vaginal tablets for 21 days	**Group A:** 64 untreated patients	LF	**IL-6 (day 30):** lower in group B vs group A (5.823 pg/ml vs 80.82 pg/ml), p < 0.0001<br><br>**Cervical length (day 30):** higher in group B vs group A (37.6 mm, [34.8 – 43.0 mm] vs 21.38 mm, [range 20.0-23.5 mm]), p < 0.0001	**No safety data provided;**<br><br>7 dropouts in the intervention group and 8 in the control group.<br>No adverse effects related to LF were reported.	

							**Relationship between CL measurements and IL-6 levels**: negative correlation in both groups (Gropu B: r = –0.81; Gropu A: r = –0.73; p < 0.0001).<br><br>**Uterine contractions** (more than six uterine contractions in 30 min): less frequent in group B (9%) vs group A (20.3%), p = 0.05		
Vesce et al. [20]<br><br>(2014)<br><br>Italy	Open-label clinical study	To evaluate the efficacy of lactoferrin in reducing IL-6 levels in amniotic fluid following amniocentesis and preventing abortion secondary to the procedure	60 pregnant patients undergoing genetic amniocentesis at the 16th gestational week	**Group B**: 20 patients treated with vaginal compound containing 300 mg of LF, 4 h before amniocentesis<br><br>**Group C**: 20 patients treated with vaginal compound containing 300 mg of LF, 12 h before amniocentesis	**Group A**: 20 untreated patients	LF	**IL-6 levels**: higher in Group A compared to Group B (1,084.1 pg/mL ± 1,458.3 pg/mL vs. 242.3 pg/mL ± 163.5 pg/mL; p = 0.03)<br><br>**IL-6 levels**: higher in Group C compared to Group B (1,315.7 pg/mL ± 1,472.2 pg/mL vs. 242.3 pg/mL ± 163.5 pg/mL; p = 0.006)<br><br>**IL-6 levels**: no significant difference was found between Group A and Group C<br><br>(1,084.1 pg/mL ± 1,458.3 pg/mL vs. 1,315.7 pg/mL ± 1,472.2 pg/mL; p = 0.5)	**No side effects observed.**<br><br>No complications reported within 7 days after amniocentesis; pregnancies proceeded to normal term delivery in all cases.	
Trentini et al. [21]<br><br>(2016)<br><br>Italy	Open-label study	To evaluate<br><br>whether treatment with LF prior to	111 pregnant women undergoing genetic amniocentesis within the 16th–	**Group B**: 54 patients treated with 300mg of vaginal LF, 4 h before amniocentesis	**Group A**: 57 untreated patients	LF	**PGE2**: lower in the group B vs group A (3.8 [2.9–6.3] pg/mg creatinine vs 5.3 [4.4–8.2] pg/mg creatinine); p < 0.01	**No side effects observed.**	Publications from the same research group; potential partial overlap of participants cannot be excluded

		amniocentesis might<br><br>influence the production of active MMP-9, active MMP-2,<br><br>their specific inhibitors, TIMP-1 and TIMP-2, and PGE2 in<br><br>the AF of pregnant women undergoing genetic<br><br>amniocentesis	18th gestational weeks				**MMP-9**: lower in group B vs. group A (39.6 [13.7–79.9] ng/mg vs 71.0 [34.5–105.0] ng/mg); $p < 0.005$<br><br>**TIMP-1**: lower in the group B (66579 [47716–80953] ng/mg vs 84048 [66029–97949] ng/mg); $p < 0.001$<br><br>**MMP-2**: higher in the group B vs. group A (270.6 [146.6–459.5] ng/mg vs 105.7 [80.6–137.2] ng/mg); $p < 0.0001$<br><br>**TIMP-2**: no difference between group (5568 [3010–9718] ng/mg vs 5098 [3277–6830] ng/mg); $p = 0.235$<br><br>**MMP-9/TIMP-1 ratio**: no difference between group (0.00022 [0.00008–0.00043] ng/mg vs 0.00025 [0.00014–0.00044] ng/mg); $p = 0.183$<br><br>**MMP-2/TIMP-2 ratio**: higher in the group B vs. group A (0.0167 [0.0104–0.0268] ng/mg vs 0.0067 [0.0050–0.0112] ng/mg); $p < 0.0001$		
Maritati et al. [25]<br><br>(2017)	Open label clinical study	To investigate AF cytokine profiles before and after	60 women undergoing genetic amniocentesis at	**Group B**: 20 patients treated with 300 mg of LF vaginal tablet 4	**Group A**: 20 untreated patients	LF	**IL-9:** reduction in Group B vs Group A, reduction in Group C vs Group A (A = 35.69 pg/mL, B = 5.89 pg/mL, C = 7.52	**No side effects observed.**<br><br>No complications reported within 7	

Italy		lactoferrin administration	the 16th gestational week	h before amniocentesis<br><br>**Group C**: 20 patients treated wtih 300 mg of LF vaginal tablet 12 h before amniocentesis			pg/mL), p < 0.001 (A vs B), p < 0.001 (A vs C)<br><br>**TNF-α:** reduction in Group B vs Group A, reduction in Group C vs Group A<br><br>(A = 61.63 pg/mL, B = 1.63 pg/mL, C = 19.99 pg/mL), p < 0.001 (A vs B), p < 0.001 (A vs C)<br><br>**IP-10:** reduction in Group B vs Group A, reduction in Group C vs Group A<br><br>(A = 38964 pg/mL, B = 7739 pg/mL, C = 8732 pg/mL), p < 0.001 (A vs B), p < 0.001 (A vs C)<br><br>**IFN-γ:** reduction in Group B vs Group A, reduction in Group C vs Group A<br><br>(A = 66.84 pg/mL, B = 24.7 pg/mL, C = 27.7 pg/mL), p < 0.001 (A vs B), p < 0.001 (A vs C)<br><br>**MCP-3:** reduction in Group B vs Group A, reduction in Group C vs Group A<br><br>(A = 174.3 pg/mL, B = 28.63 pg/mL, C = 30.49 pg/mL), p < 0.001 (A vs B), p < 0.001 (A vs C)	days after amniocentesis; all pregnancies proceeded normally to term delivery.	

							**IL-15:** reduction in Group B vs Group A, reduction in Group C vs Group A<br><br>(A = 81.27 pg/mL, B = 0.42 pg/mL, C = 0.42 pg/mL), $p < 0.01$ (A vs B)<br><br>**IL-1α:** reduction in Group B vs Group A, reduction in Group C vs Group A<br><br>(A = 7.9 pg/mL, B = 0.35 pg/mL, C = 0.35 pg/mL), $p < 0.05$ (A vs B), $p < 0.001$ (A vs C)<br><br>**IL-2RA:** reduction in Group B vs Group A, increase in Group C vs Group A<br><br>(A = 1659 pg/mL, B = 800.9 pg/mL, C = 5957 pg/mL), $p < 0.01$ (A vs B)<br><br>**IL-12p40:** reduction in Group B vs Group A, reduction in Group C vs Group A<br><br>(A = 1809 pg/mL, B = 1.17 pg/mL, C = 1.17 pg/mL), $p < 0.01$ (A vs B)<br><br>**IFN-α2:** reduction in Group B vs Group A, reduction in Group C vs Group A<br><br>(A = 195.1 pg/mL, B = 127.3 pg/mL, C = 120.1 pg/mL), $p < 0.05$ (A vs B)		

							**IL-2:** reduction in Group B vs Group A, reduction in Group C vs Group A<br><br>(A = 30.04 pg/mL, B = 0.2 pg/mL, C = 0.2 pg/mL), $p < 0.05$ (A vs B)<br><br>**IL-4:** reduction in Group B vs Group A, reduction in Group C vs Group A<br><br>(A = 1.97 pg/mL, B = 0.06 pg/mL, C = 1.09 pg/mL), $p < 0.05$ (A vs B)<br><br>**EOTAXIN:** reduction in Group B vs Group A, reduction in Group C vs Group A<br><br>(A = 300.4 pg/mL, B = 6.01 pg/mL, C = 15.67 pg/mL), $p < 0.05$ (A vs B)<br><br>**PDGF-BB:** reduction in Group B vs Group A, reduction in Group C vs Group A<br><br>(A = 89.13 pg/mL, B = 39.86 pg/mL, C = 50.43 pg/mL), $p < 0.05$ (A vs B)<br><br>**RANTES:** reduction in Group B vs Group A, reduction in Group C vs Group A<br><br>(A = 37.75 pg/mL, B = 0.52 pg/mL, C = 21.37 pg/mL), $p < 0.05$ (A vs B)		

							**IL-18:** reduction in Group B vs Group A, reduction in Group C vs Group A<br><br>(A = 240 pg/mL, B = 98.97 pg/mL, C = 67.72 pg/mL), $p < 0.05$ (A vs C)<br><br>**MIF:** reduction in Group B vs Group A, reduction in Group C vs Group A<br><br>(A = 2155 pg/mL, B = 1440 pg/mL, C = 1446 pg/mL), $p < 0.05$ (A vs B), $p < 0.05$ (A vs C)<br><br>**IL-17:** reduction in Group B vs Group A, increase in Group C vs Group A<br><br>(A = 37.02 pg/mL, B = 0.44 pg/mL, C = 302.8 pg/mL), $p < 0.05$ (A vs B), $p < 0.001$ (A vs C), $p < 0.001$ (B vs C)<br><br>**FGF-b**: reduction in Group B vs Group A, increase in Group C vs Group A<br><br>(A = 36.59 pg/mL, B = 0.25 pg/mL, C = 100 pg/mL), $p < 0.001$ (A vs B), $p < 0.05$ (A vs C), $p < 0.001$ (B vs C)<br><br>**G-CSF:** increase in Group B and C vs Group A<br><br>(A = 115.5 pg/mL, B = 162 pg/mL, C = 742.1 pg/mL), $p < 0.001$ (A vs B), $p < 0.01$ (A vs C)		

							**GM-CSF**: increase in Group B and C vs Group A<br><br>(A = 231 pg/mL, B = 300 pg/mL, C = 350.5 pg/mL), $p < 0.01$ (A vs B)<br><br>**MCP-1:** increase in Group B and C vs Group A<br><br>(A = 167.8 pg/mL, B = 401.2 pg/mL, C = 374.6 pg/mL), $p < 0.05$ (A vs C)<br><br>**IL-3:** increase in Group B and C vs Group A<br><br>(A = 404.7 pg/mL, B = 537.7 pg/mL, C = 537.7 pg/mL), $p < 0.05$ (A vs B)<br><br>**SDF-1α:** increase in Group B and C vs Group A<br><br>(A = 152 pg/mL, B = 541 pg/mL, C = 617 pg/mL), $p < 0.05$ (A vs C)		
Miranda et al. [24]<br><br>(2019)<br><br>Italy	Retrospective study	To evaluate use of vaginal lactoferrin in prevention of PTB in women with first trimester bacterial vaginosis and prior spontaneous<br><br>PTB	125 pregnant women with singleton gestations and history of prior spontaneous PTB and bacterial vaginosis, at $16^{0/7}$–$36^{6/7}$ weeks of gestation	**Group B**: 60 women with BV who received 300 mg vaginal lactoferrin tablets daily, for 21 days	**Gropu A**: 65 women with BV,<br><br>untreated	LF	**PTB < 37 weeks**: lower in the group B vs group A (25.0% vs 44.6%), $p = 0.02$<br><br>**Mean Gestational Age at Delivery:** higher in the group B vs group A (37.7 ± 3.2 weeks vs 35.9 ± 4.1 weeks), $p = 0.01$	**No side effects observed.**	

							**Late miscarriage:** no cases reported in either Group A or B<br><br>**Hospitalization for PTL:** less frequent in the group B vs group A (45.0% vs 70.8%), p = 0.04<br><br>**Chorioamnionitis:** no significant differences between groups, p=0.27<br><br>**Preterm premature rupture of membranes (PPROM < 34 weeks):** no significant differences between groups, p=0.53<br><br>**PPROM < 34 weeks:** less frequent in the group B vs group A (1.7% vs 3.1%), p =0.53<br><br>**Birth weight:** higher in the group B vs group A (2,808 ± 719 g vs 2,625 ± 831 g), p = 0.19<br><br>**NICU admission:** lower in the group B vs group A (8.3% vs 21.5%), p = 0.05		
Trentini et al. [23]<br><br>(2020)	Open-label clinical study	To determine whether vaginal LF administration is able to decrease OxS	60 pregnant women undergoing genetic amniocentesis at	**Group B**: 20 patients treated with 300mg of vaginal LF, 4 h before amniocentesis	**Group A**: 20 untreated patients	LF	**TBARS levels**: lower in group B and C vs group A (Gropu B vs. Group A: p < 0.0001, and in Group C vs. Group A: p < 0.05)	**No side effects observed.**	Publications from the same research group; potential partial overlap of participants

Italy		measured in the amniotic fluid of pregnant women undergoing genetic amniocentesis	16th gestational week	**Group C**: 20 patients treated with 300mg of vaginal LF, 12 h before amniocentesis			No significant difference between Group B and Group C, $p = 0.416$<br><br>**TAS**: increased in the Group B: 35% vs Group A, $p < 0.0001$; 17% higher than group C, $p < 0.001$<br><br>No significant difference between Group C and Group A<br><br>**OSI:** lower in the Group B vs Group A, $p < 0.0001$		cannot be excluded

Abbreviations: LF: lactoferrin; rhLf: recombinant human lactoferrin; bLF: bovine lactoferrin; TAS: Total Antioxidant Status; OSI: Oxidative Stress Index; PGE2: Prostaglandin E2; MMP: matrix metalloproteinase; TIMP: Tissue inhibitor of metalloproteinase; AF: Amniotic Fluid; PTB: prevention of preterm birth; PTD: preterm delivery; BV: bacterial vaginosis; OxS: Oxidative Stress; NR: not reported; ID: Iron deficiency; IDA: iron deficiency anemia.
